# Micro-Finned Nanocomposite Films for Enhanced Transport Properties: Graphite Nanoplatelet-Filled Linear Low-Density Polyethylene

**DOI:** 10.3390/polym15224411

**Published:** 2023-11-15

**Authors:** Sagar V. Kanhere, Özgün Güzdemir, Amod A. Ogale

**Affiliations:** Center for Advanced Engineering Films and Fibers (CAEFF), Department of Chemical and Biomolecular Engineering, Clemson University, Clemson, SC 29634, USA; skanher@clemson.edu (S.V.K.);

**Keywords:** graphite nanoplatelets, thermal conductivity, electrostatic dissipation, microtextured films

## Abstract

Metals are being replaced with high-performance and lightweight polymers, but their low thermal conductivity and poor electrostatic dissipative properties are significant problems. For the protection of sensitive electronic circuitry in automotive and aerospace parts, some device housing materials must provide electrostatic discharge and dissipate heat generated at higher rates as electronic circuits are increasingly miniaturized. Micro-texturing on the film surface can greatly enhance the heat dissipation area and was investigated in this study using low-cost graphite nanoplatelet (GNP)-filled LLDPE films. Micro-finned films (30 vol% GNP) having a 51 ± 10% larger heat-dissipation area were successfully produced using a continuous extrusion process. The through-thickness thermal conductivity of 30 vol% GNP-filled LLDPE was measured at 1.3 W/m·K, which represents a 200% improvement over that of pure LLDPE. For a GNP content of 30 vol%, the surface and volume electrical conductivity of the composite films also increased by 8 orders of magnitude (resistivity down from ≈10^15^ to 10^7^ Ω·cm) and electrostatic decay time reduced to a below-resolution limit of 0.01 s, at par with military standard requirements. Thus, micro-fin textured GNP-LLDPE offers a unique combination of electrical and thermal transport desired for the protection of electronic encapsulation materials.

## 1. Introduction

Polymer materials are widely used in consumer electronics critical to the operations of vehicles, satellites, airplanes, and as well as other weight, cost, and corrosion-sensitive applications. Polymers have low density, versatility regarding forming into different shapes, and corrosion resistance. Most polymers, however, are electrically and thermally insulating. In contrast, metals are electrically and thermally conducting but possess high density and are highly susceptible to corrosion.

As the number of high-frequency miniaturized electronic devices performing at higher speeds increases, significant heat is generated during processing, and instruments are increasingly vulnerable to static discharge and heat accumulation. To protect the instrument from damage due to internal heat buildup, the packaging material must be efficient for heat dissipation to retain the performance of the electronic part. Electrostatic buildup occurs due to charge separation in a non-conducting material, which results in intense electric fields. The electrostatic charge that builds up on electronic packaging can not only interfere with radio communication, which can have catastrophic consequences, but also cause sparks that can lead to fire hazards. To protect the electronic part from static discharge surges, packaging material must also be able to protect the electronic component from outside electrical activity and should dissipate electrostatic charge buildup [1,2]. Finally, electronic packaging materials used in aerospace and automotive parts are also expected to be lightweight for fuel economy and enhance consumer experience.

Traditionally, metals and/or ceramics are used as heat sinks, which are not only expensive and heavy, but are also highly susceptible to cracking and cannot sustain severe vibrations [3]. Therefore, polymer composites with thermally and electrically conducting fillers are investigated as potential replacements alternatives for metal parts. Metal powders, carbon nanotubes, carbon fibers, and graphite powder have been investigated as fillers to improve the transport properties of composite materials [4,5,6,7,8,9]. Since most metal powders have high density, and to achieve the desired properties, high-volume fractions of the powder need to be mixed with polymer, there is very little weight savings, if any. Carbon nanotubes or carbon fibers are a prohibitively expensive reinforcement for most applications [10,11,12].

Exfoliated graphite nanoplatelets (GNPs), short stacks of graphene sheets, are a cheaper alternative to fillers like carbon nanotubes for producing such composites [2]. GNPs are thermally and electrically conducting [11]. Yu et al. [13] studied GNP-reinforced epoxy composites for thermal management and reported significant improvement in the thermal conductivity of the composites with 25 vol% filling. However, the material was processed on a lab scale, and it is unclear whether it can be made for large-scale applications. A uniform dispersion of the graphene in the polymer matrices has been an issue due to the strong van der Waals interactions, and interplanar π–π interactions make it challenging to disperse them in the matrices [12]. Han et al. [14] studied the relationship between the microstructure and properties of graphene/polyethylene composites and found that the volume fraction and interfacial interaction have a strong influence on the thermal and mechanical properties of the resulting composites. Kim et al. [15] coated GNPs with paraffin to improve dispersion in linear low-density polyethylene (LLDPE) nanocomposites. The composites prepared by the solution method showed very high resistivity (10^11^ ohm-cm). However, melt mixed composites resulted in less resistivity (10^5^ ohm-cm). Moreover, Sun et al. [4] investigated that the thermal conductivity of epoxy/10 wt% GNP nanocomposites filled with three GNPs increases with increasing aspect ratio. Hing et al. [5] showed that better interfacial bonding slightly improved the thermal conductivity of epoxy/GNP composites as well as the mechanical properties. When they treated GNPs with nitric acid, the thermal conductivity of the composites increased from 0.21 W/m·K to 0.23 W/m·K. Huang et al. prepared hyper-branched aromatic polyamide (HAP)-grafted h-BNNPs and then epoxy/BN nanocomposites. Hyper-branched aromatic polyamide-grafted on carbon nanotubes improved the thermal conductivity by 25% and it increased to 1 W/m·K [6].

The micro-texturing of the films creates an extended surface area that can improve the heat dissipation rate. Typically, the micro-texturing of films is performed using chemical etching, photolithography, mold casting, or laser texturing, but all those processes are batch processes and are time-consuming [16,17]. In our prior study [18], microtextured boron nitride-filled LLDPE composite films were produced using a continuous twin-screw extruder that exhibited increased heat dissipation area, but boron nitride is electrically insulating. Thus, none of the prior studies have explored the effect of micro-texturing on the combined thermal conductivity and electrostatic dissipation characteristics, which are the focus of this study.

## 2. Materials and Methods

### 2.1. Materials and Film Processing

**Figure 1:**
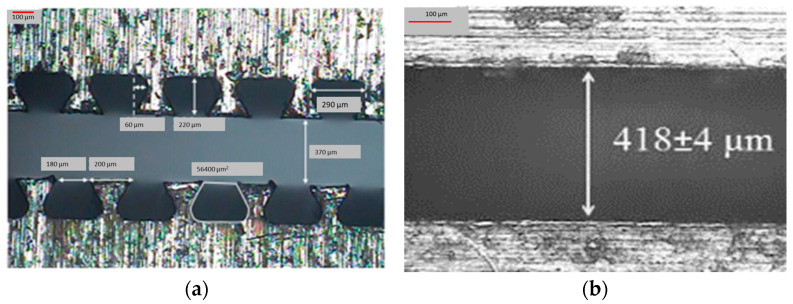
Two different dies showing: (**a**) trapezoidal-shaped micro-pattern for textured films, and (**b**) nontextured die.

A linear low-density polyethylene (Aspun 6835, Dow Chemical Company, Midland, MI, USA) was used as the matrix throughout this study. The polymer had a density of 0.95 g/cc, a melting point of 129 °C, and a melt flow index of 17 g/10 min. Graphite nanoplatelets (GNPs) of Nano25 grades (Asbury Carbons, Inc., Asbury, NJ, USA) were used as fillers. GNP-filled LLDPE films were prepared through melt-extrusion using a co-rotating twin-screw extruder MC1HT micro-extruder (DSM Xplore, Geleen, The Netherlands). For this study, melt-compounding of LLDPE and GNP powder was performed by allowing 15 min of mixing time at 210 °C. Textured films were extruded through a die with a trapezoid-shaped cavity with distance between protruding features of nominally 370 µm (see Figure 1a), whereas nontextured films were extruded using a die with rectangular cavity as shown in Figure 1b. Films were extruded at 210 °C, which is well below the degradation temperature of 250 °C. The thermogravimetric analysis of the films was conducted using Pyris 1 TGA (Perkin Elmer, Waltham, MA, USA). For each test, at least three replicates were measured.

Films extruded through both dies were 32 mm wide. For preparing films of larger size, which are required for electrostatic dissipation (ESD) and resistivity measurements, films were pressed together using a hot hydraulic press (Carver^®^ Inc., Wabash, IN, USA) under a 24 kN force at 110 °C to produce the required film size.

### 2.2. Material and Film Characterization

To analyze the carbon structure of GNPs, the powder was scanned under Renishaw inVia Raman microscope, using a laser wavelength of 785 nm and laser power of 25 mW. The laser was focused on the platelet surface using 50× magnification. Data were analyzed using WiRE Raman Software version 3.4. A silicon standard with Raman shift at 520 cm^−1^ was used to calibrate the Raman detector.

Scanning electron microscopy images of GNPs and films were obtained using Hitachi Regulus scanning electron microscope (SEM) to analyze their microstructure. For SEM imaging, LLDPE films were coated with platinum, whereas GNPs and GNP-filled films do not need coating due to their conducting nature. The accelerating voltage used during imaging was set between 5 and 10 kV, and the beam current was 10 µA.

Viscosity measurements of the unfilled and filled polymers were performed at 210 °C (i.e., identical to the extrusion temperature) using a TA Instruments ARES rheometer with a parallel-plate fixture (D = 25 mm). Extruded film samples were pressed to form a disc and re-melted to characterize the viscosity. Tensile properties were measured following the ASTM D638-14 standard [19] using ATS universal 900 tensile tester (Applied Test Systems, PA, USA).

A laser flash analysis (LFA) technique (LFA447 Nanoflash, Netzsch, Germany) was used to measure the through-thickness thermal conductivity of the GNP/LLDPE films by following the ASTM E1461-13 standard [20] test method. Square specimens of 10 × 10 × 0.2 mm were prepared for through-thickness thermal conductivity measurements. Three replicate specimens were analyzed per composite composition and film type, and three repeated measurements were carried out per specimen. Through-thickness thermal diffusivity values were calculated using the Cowan + pulse correction model that accounts for possible heat losses during measurement. Thermal diffusivity values for micro-textured films were calculated using equivalent thickness calculated by dividing the cross-sectional area by the width of the film from the cross-sectional images of the films.

The surface and volume resistivity of the specimen were measured with a Keithley 6517B Electrometer (Textronics Company, Cleveland, OH, USA) using Model 8009 resistivity test fixture by following ASTM D257 standard [21]. Electrostatic decay time was measured following MIL-STD-3010C test method 4046 [22] using Static Decay Meter Model 406D manufactured by Electro-Tech Systems, Inc. (Glenside, PA, USA). The decay time readout has a resolution of 0.01 s. Relative humidity was maintained at less than 12% during measurements, and specimens were conditioned for 24 h at 25 °C. Commercial antistatic ESD-compliant electronic packaging film was used as control, and its decay time was measured to be 0.01 s at a 1% cut-off when charged to 5 kV.

## 3. Results

### 3.1. Processing and Microstructure

#### 3.1.1. Melt Rheology

**Figure 2:**
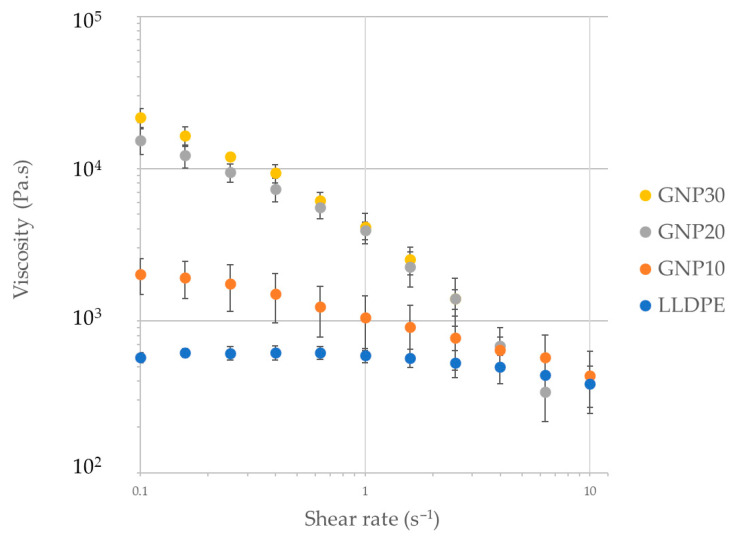
Shear viscosity of the GNP/LLDPE-filled melt mixtures.

To assess the role of solid particulates on the flow during film extrusion, steady and dynamic shear viscosity was measured. Figure 2 displays the steady shear viscosity of the GNP/LLDPE mixture over a range of shear rates measured using a parallel plate. As expected, the shear viscosity of the melt increases with increasing GNP content in the polymer. More than half an order of magnitude increase in shear viscosity was observed at 10 vol% GNP filling, whereas it increased by 1.5 orders of magnitude for 30 vol% filling as compared to pure polymer at a shear rate of 0.1 s^−1^. This increase in shear viscosity was generally consistent with that estimated using Mooney’s equation [23,24]. However, as the shear rate increases, the viscosity of GNP-LLDPE decreases more significantly (i.e., a greater shear thinning behavior). This behavior arises from the flow-alignment of anisotropic particles (GNPs are platelets) and they tend to flow in parallel to the nearest die surface, consistent with similar observations from prior studies in the literature [18,25,26].

Table 1 displays the power-law fitting parameters (Equation (1)) for the shear viscosity of the GNP/LLDPE mixture melts. As GNP content increased, the power law exponent decreased, which confirms the increasing shear thinning behavior of the GNP-filled LLDPE melt.

(1) $$\eta =K{\dot{\gamma }}^{n-1}$$

where *η*—viscosity, $\dot{\gamma }$—shear rate, *n*—power law exponent, *K*—power law index.

**Figure 3:**
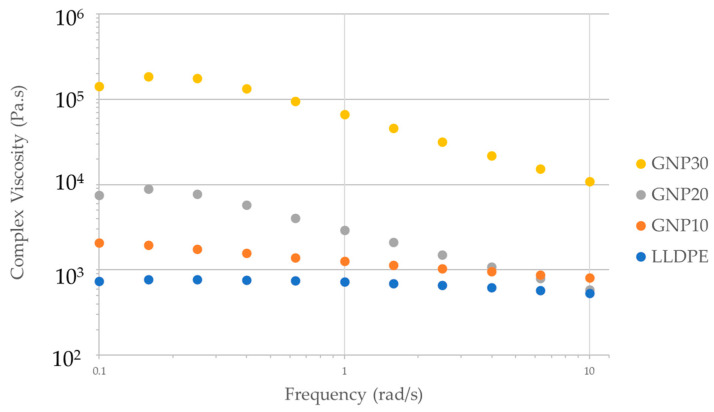
Complex viscosity of the BN/LLDPE melt at 210 °C at different GNP content.

Dynamic frequency sweep measurements were conducted on the GNP-filled LLDPE melt between 0.1 and 10 rad/s. Small amplitude oscillation offers insight into the inherent viscoelastic nature of the polymer melt, in contrast to that observed due to particle flow-alignment during steady shear flow. Figure 3 displays complex viscosity as a function of angular frequency. As compared to that of pure LLDPE, the complex viscosity of the 30 vol% GNP-filled LLDPE was measured to be two orders of magnitude higher at 0.1 rad/s, a consistent trend with steady shear viscosity measurements. It was observed that the loss and storage modulus of the melt increases with an increase in frequency, which is consistent with observations in prior studies of nanocomposites melt [27].

#### 3.1.2. Graphite Nanoplatelets Characteristics

**Figure 4:**
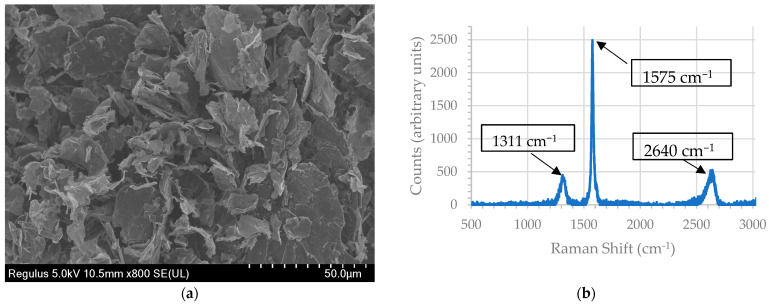
Graphite nanoplatelet characteristics: (**a**) Scanning electron micrograph; (**b**) Raman spectra.

A representative SEM micrograph of GNPs is displayed in Figure 4a. Using qualitative image analysis, it was found that GNP platelets were about 300–600 nm thick with diameters ranging up to 50 μm. Raman spectra of the GNP are shown in Figure 4b. The D peak is an indication of disordered carbon, whereas the G peak is an indication of graphitic carbon. G′ is a secondary resonance peak indicative of the stacking of the carbon layers. The I(D)/I(G) ratio is the ratio of the area under the curve of D and G peaks, which represents the ratio of disordered to ordered carbon in the GNP. Raman spectra analysis of GNPs found that the D peak was at 1311 cm^−1^, the G peak was at 1575 cm^−1^, and the G′ peak was at 2640 cm^−1^. For GNP platelets, the I(G)/I(D) ratio was measured at 0.5, which is consistent with prior literature studies [28]. Thus, GNPs have a highly ordered carbon structure, which is essential to make GNPs highly electrically and thermally conducting.

#### 3.1.3. Film Microstructure

**Figure 5:**
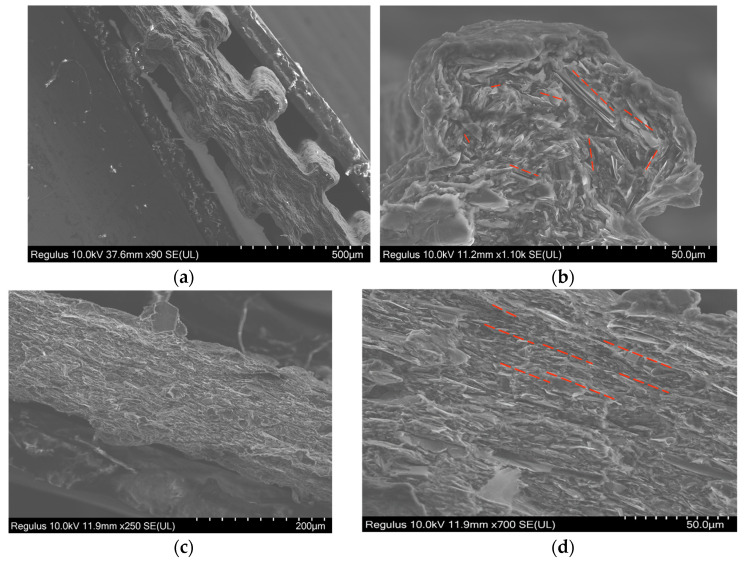
Scanning electron microscopy images of cross-section of 30 vol% GNP-filled films: textured films (**a**,**b**) and nontextured films (**c**,**d**). Red dashed lines indicate the orientation of GNP platelets.

Scanning electron microscopy (SEM) micrographs of textured and nontextured films are displayed in Figure 5 (the aforementioned Figure 1 displays cross-sections of textured and nontextured dies). The nontextured film thickness was about 200 µm, and the graphite nanoplatelets were oriented in the extrusion direction. The textured films have a similar base thickness of 150 µm, but have added texture heights of about 50 to 100 µm (Figure 5a). The nanoplatelets in the extended textures are parallel to the nearest die surface, as shown in Figure 5b. This orientation is attained due to shear stresses exerted in the melt by the die during film extrusion. The GNP content is similar in micro-fins and the bulk of the film.

**Figure 6:**
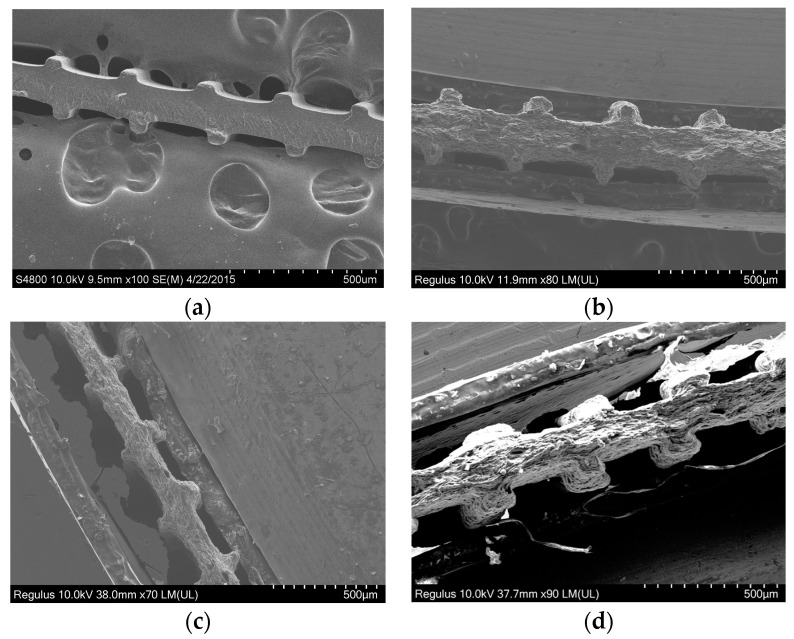
Microtextured films with GNP content of (**a**) 0 vol% (i.e., pure LLDPE); (**b**) 10 vol%; (**c**) 20 vol% and (**d**) 30 vol %.

SEM micrographs of the textured film cross-section are displayed in Figure 6. As evident from Figure 6a, the textured LLDPE film has the extended surfaces shaped as trapezoidal micro-fins. The length of the outer side of the fin is 44 ± 3 µm, which is smaller than the base of 66 ± 2 µm that is attached to the bulk of the LLDPE film. In contrast, for 30 vol% GNP-filled textured film, the length of the inside base attached to the base of the film is slightly smaller than the outer side to yield an inverted trapezium similar to the die shape (i.e., the inside length slightly less and outside slightly more than 100 µm). Because of the viscoelasticity of the polymeric melt, the extrudates do not retain the trapezoidal shape of the die, instead attain the texture shown in the representative SEM micrograph displayed in Figure 6a. As the GNP filling content increased, the relaxation time of the polymer melt rises due to decreased molecular elasticity, which makes the shape of the micro-fins resemble more like the trapezoidal shape of the die, as evident from Figure 6d.

For convective cooling, the lateral surface (i.e., textured surface area) would be exposed to the cooling fluid, and the cooling rate is U.A.ΔT, where U is the overall heat transfer coefficient, A is area of heat transfer, and ΔT is the temperature difference driving the heat transfer. Therefore, an increased surface area enhances the convective heat transfer rate proportionally. Lateral surface area is the product of the perimeter (as measured in the cross-section) and the length (same for all films). Therefore, the increase in the perimeter due to micro-fins directly represents the increase in the area. Based on the measured perimeters of the film cross-sections, these extended micro-finned textures provide about 16 ± 3, 20 ± 4, 26 ± 5 and 51 ± 10% greater extended surface area for heat dissipation as compared to their nontextured counterparts (for the same nominal width) for films containing 0 (i.e., pure LLDPE), 10, 20 and 30 vol% GNP content, respectively. Thus, micro-texturing directly increases the area of heat transfer and, consequently, overall heat dissipation rate.

### 3.2. Thermogravimetric Analysis of the Film

**Figure 7:**
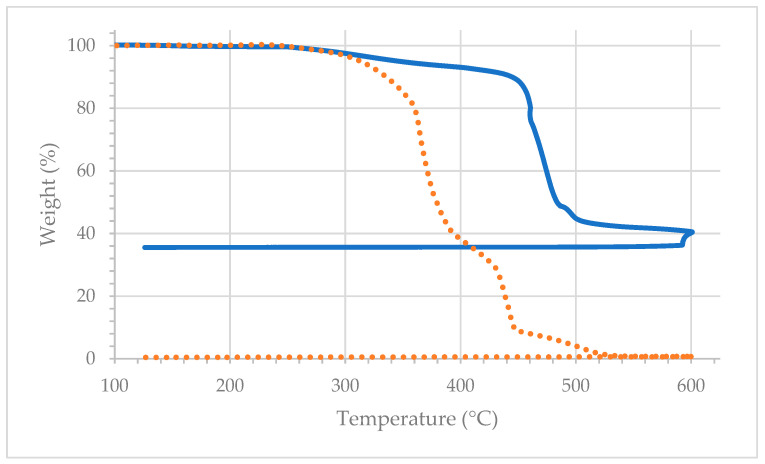
Thermogravimetric analysis of LLDPE (dotted line) and 30 vol% GNP-filled LLDPE films (solid line) in the air.

Figure 7 displays the thermogravimetric analysis of LLDPE and 30 vol% GNP-filled films in air. It was observed that both films start to thermo-oxidatively degrade at approximately 250 °C. LLDPE film rapidly degrades above 350 °C, whereas GNP-filled film exhibits a slower degradation until approximately 450 °C, as evident from the smaller slope of the thermogram (between 350 and 450 °C). Thus, the onset degradation temperature remained at 250 °C for both grades, i.e., it did not change significantly due to the addition of GNPs. Note that the extrusion temperature used throughout this study (210 °C) is well below the onset degradation temperature.

### 3.3. Mechanical Analysis

**Figure 8:**
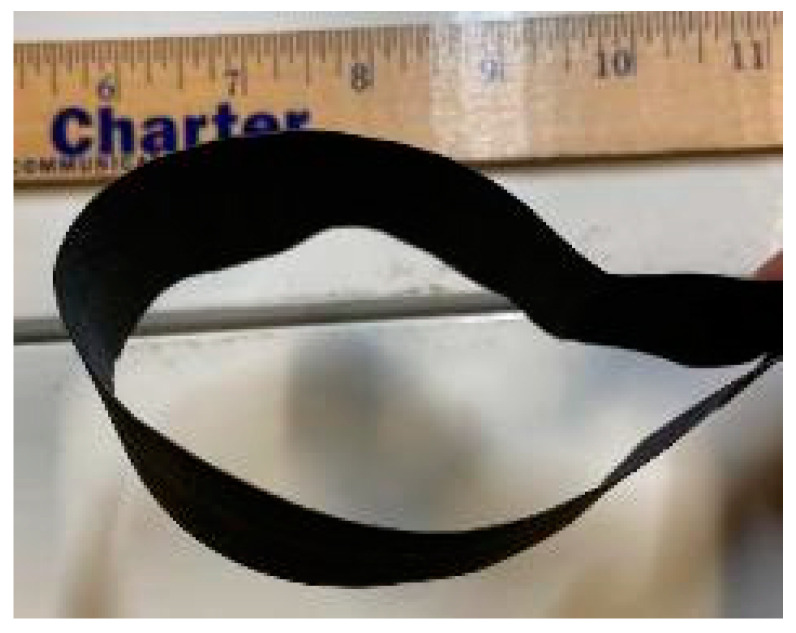
A 30 vol% GNP-LLDPE film shown folded upon itself.

Figure 8 displays a 30 vol% GNP-LLDPE film that could be folded upon itself. Even after 30 vol% GNP filling, the textured and nontextured films remained flexible enough to bend without fracture. The tensile strength was measured at 23 ± 0.4 MPa for neat LLPDE film and 10 ± 3 MPa for 30 vol% GNP-filled LLDPE. This drop is typical of discontinuous non-oriented fillers, which almost always reduce in strength. In contrast, tensile modulus increased from 456 ± 23 MPa for pure LLDPE to 900 ± 110 MPa for 30 vol% GNP-filled LLDPE. Again, this increase in modulus is typical for all composites, including particulate and discontinuous inclusions.

### 3.4. Thermal Conductivity

**Figure 9:**
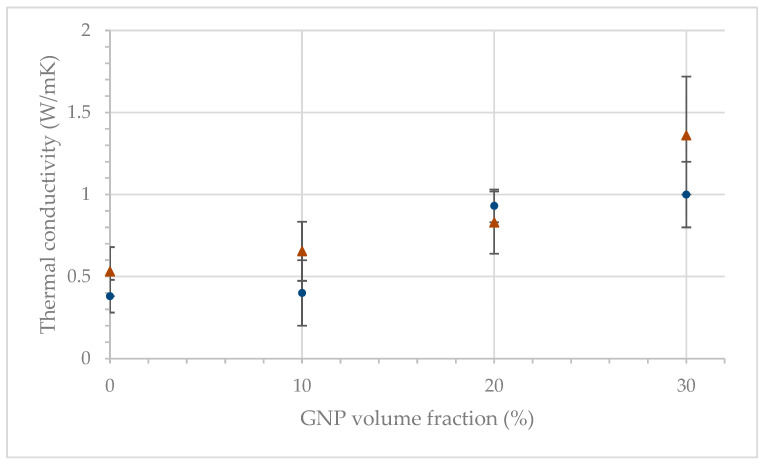
Thermal conductivity of the textured (Δ) and nontextured (●) GNP-filled LLDPE films at different GNP contents.

Figure 9 shows the through-thickness thermal conductivity of the GNP nanocomposite films at various GNP volume fractions. Through-thickness thermal conductivity for pure LLDPE was measured to be about 0.4 W/m.K, consistent with prior reported results of polyethylene conductivity [29,30]. With an increase in GNP volume fraction, thermal conductivity of the nanocomposite increased for both nontextured and textured films. The thermal conductivity of the composite increased by 200% at 30 vol% filling content for both textured and nontextured films. This trend is consistent with prior observations with different polymer-filled GNP films and this enhancement in thermal conductivity is achieved due to phonon transfer facilitated by GNP fillers [13,30].

Results also show that there is no statistically significant difference between the thermal conductivity of textured and nontextured films. Thus, the textured geometry did not deteriorate the thermal conductivity of the GNP-filled LLDPE films.

### 3.5. Volume and Surface Resistivity

**Figure 10:**
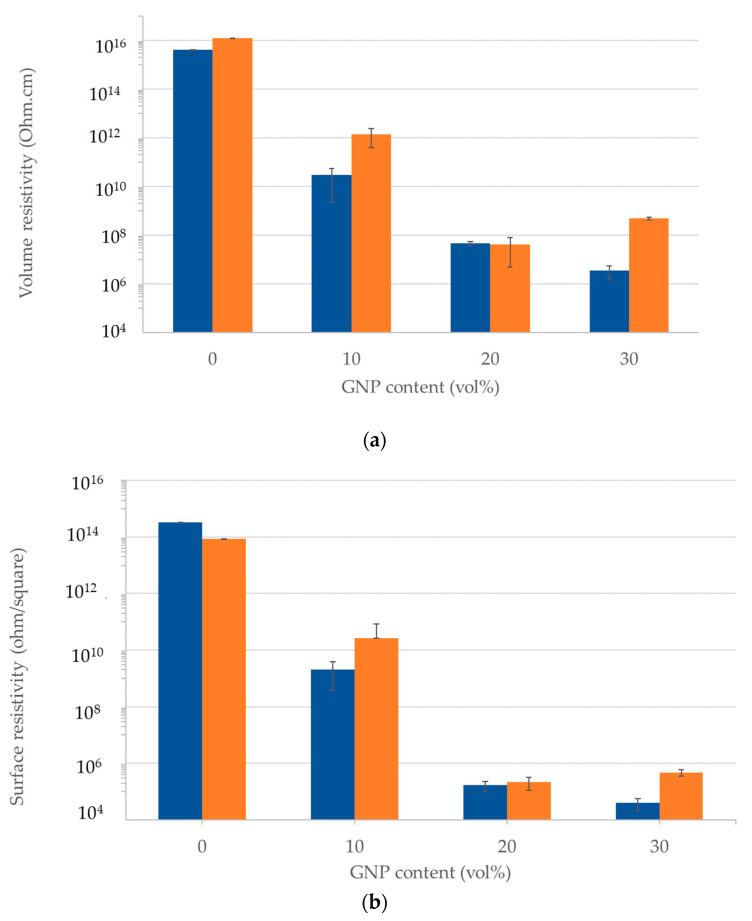
Electrical properties of textured (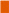) and nontextured (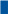) GNP-filled LLDPE films: (**a**) Surface resistivities and (**b**) volume resistivities.

Figure 10a shows the volume electrical resistivity of GNP-filled nanocomposites. Pure polyethylene volume resistivity magnitude was measured at 10^16^ Ohm.cm for both textured and nontextured films. This value is generally consistent with prior reported values [31]. With the addition of 10 vol% GNPs in LLDPE, the volume resistivity decreased exponentially by more than six orders of magnitude. This trend continued with a further increase in GNP content, with 30 vol% GNP nanocomposite films having resistivity lower by eight to ten orders of magnitude as compared with that of pure LLDPE. Because GNP platelets are significantly more conductive as compared to the polymer, upon the addition of GNP platelets to the polymer, electron transport is facilitated by the GNPs, which leads to a drastic reduction in electrical resistivity. This decrease in electrical resistivity is consistent with the prior reports of GNP-filled nanocomposites [30].

The results also show that there was no statistically significant difference between resistivities of the textured and nontextured films. Thus, the micro-texturing of the film surface did not lead to the deterioration of electrical resistivity properties of the GNP-filled nanocomposites. Overall, the nanocomposite material remains fairly conductive as compared to pure polymer films.

Figure 10b shows the surface resistivity of the nanocomposite film at different GNP filling contents. Surface resistivity is a measure of the leakage of current along the surface of the insulating material. The surface resistivity of the LLDPE was measured at 10^14^ Ohm/square, consistent with prior reports [31]. Surface resistivities decreased drastically with increasing GNP content, following the same trend as that of volume resistivities. Micro-finned surface did not impact the surface resistivities of the nanocomposite films. However, as expected, GNP addition significantly reduced the surface resistivities of the GNP-filled LLDPE, which is desired for static charge dissipation.

### 3.6. Electrostatic Decay Time

Table 2 displays the electrostatic dissipation time for films analyzed in this study. For calibration purposes, a commercial antistatic-compliant bag was tested, and 1% cut-off time was found to be below the detection limit of 0.01 s. For neat LLDPE films, the decay time for 1% cut-off was measured to be 531 s and 17 s for nontextured and textured films, respectively. This difference may be because the amount of charge buildup depends on the shape, and an extended surface provides more opportunities for charge dissipation [32].

With the addition of 10 vol% GNP, ESD decay time reduced drastically to 1.3 s for nontextured film, whereas it was 0.02 s for micro-finned textured film. The 20 and 30 vol% GNP-filled LLDPE films showed an ESD decay time at the lower measurement limit of the instrument.

Per military standard Mil-B-81705C and Mil-B-81705B [33,34], 99% of the static charge buildup (i.e., 1% cut-off) on the surface must be dissipated in less than 2 s. Surface conductivity of the GNP-filled LLDPE films increased significantly with the addition of GNP, which creates static charge dissipation pathways [35]. Further, the extra surface created via micro-finned texturing did not deteriorate surface resistivity, but rather enhanced ESD characteristics. Overall, GNP-filled films with 10 vol% GNPs have an ESD decay time of less than 2 s, which reduced to an immeasurably low time of under 0.01 s at 30 vol% GNP content. Thus, GNP-filled films have the potential as RoHS-compliant ESD shielding packaging at 20 and 30 vol% GNP. In summary, GNP filler not only improved the thermal conductivity of the films, but also significantly improved the electrical conductivity and ESD characteristics of the films, and the texturing increased the area of heat dissipation by 50 ± 10% without adversely impacting any of the electrical characteristics of the film.

## 4. Conclusions

This study demonstrated that the GNP-filled LLDPE films with a significant extended surface area provided by micro-fins can be successfully produced using a continuous extrusion process. The micro-fins led to about a 51 ± 10% increased area for heat dissipation, and 30 vol% GNP content led to a 200% increase in thermal conductivity of the films when compared with pure LLDPE films. This combination of enhanced thermal conduction and convection effects is a powerful combination to enhance the overall rate of heat dissipation. The electrical conductivity of the composites increased significantly with GNP content, which is reflected in the electrostatic decay time being less than 2 s for all GNP-filled composites, making them ESD compliant for most electronics applications. Thus, the current results establish that micro-finned GNP-filled LLDPE films possess a unique combination of enhanced heat dissipation and superior ESD discharge characteristics that are desired transport properties for electronic encapsulation applications.

## Figures and Tables

**Table 1:** Power law fitting parameters of the shear viscosity measurements of the GNP-filled LLDPE melt at 210 °C.

GNP Volume Fraction (%)	Power-Law Exponent, *n*	Power-Law Flow Index, Pa.s^n^
0	0.91	544
10	0.66	1037
20	0.11	2754
30	0.17	3719

**Table 2:** Electrostatic dissipation characteristics of textured and nontextured linear low-density polyethylene (LLDPE) films filled with various contents of graphite nanoplatelets (GNPs).

Material	Average 1% Cut-Off Average Decay Time (s)	
RoHS-compliant ESD shielding bag	Below Detection Limit (≤0.01 s)	
GNP content (vol%)	Nontextured	Textured
0	531	17
10	1.29	0.02
20	below detection limit	below detection limit
30	below detection limit	below detection limit

## Data Availability

The data presented in this study are available on request from the corresponding author.
